# Membrane-Modulating Drugs can Affect the Size of Amyloid-*β*_25–35_ Aggregates in Anionic Membranes

**DOI:** 10.1038/s41598-018-30431-8

**Published:** 2018-08-17

**Authors:** Adree Khondker, Richard J. Alsop, Sebastian Himbert, Jennifer Tang, An-Chang Shi, Adam P. Hitchcock, Maikel C. Rheinstädter

**Affiliations:** 1 Department of Physics and Astronomy, McMaster University, Hamilton, Ontario Canada; 2 Origins Institute, McMaster University, Hamilton, Ontario Canada; 3 Department of Chemistry and Chemical Biology, McMaster University, Hamilton, Ontario Canada

## Abstract

The formation of amyloid-*β* plaques is one of the hallmarks of Alzheimer’s disease. The presence of an amphiphatic cell membrane can accelerate the formation of amyloid-*β* aggregates, making it a potential druggable target to delay the progression of Alzheimer’s disease. We have prepared unsaturated anionic membranes made of 1-palmitoyl-2-oleoyl-sn-glycero-3-phosphocholine (POPC) and 1,2-dimyristoyl-sn-glycero-3-phospho-L-serine (DMPS) and added the trans-membrane segment A*β*_25–35_. Peptide plaques spontaneously form in these membranes at high peptide concentrations of 20 mol%, which show the characteristic cross-*β* motif (concentrations are relative to the number of membrane lipids and indicate the peptide-to-lipid ratio). We used atomic force microscopy, fluorescence microscopy, x-ray microscopy, x-ray diffraction, UV-vis spectroscopy and Molecular Dynamics (MD) simulations to study three membrane-active molecules which have been speculated to have an effect in Alzheimer’s disease: melatonin, acetylsalicyclic acid (ASA) and curcumin at concentrations of 5 mol% (drug-to-peptide ratio). Melatonin did not change the structural parameters of the membranes and did not impact the size or extent of peptide clusters. While ASA led to a membrane thickening and stiffening, curcumin made membranes softer and thinner. As a result, ASA was found to lead to the formation of larger peptide aggregates, whereas curcumin reduced the volume fraction of cross-*β* sheets by ~70%. We speculate that the interface between membrane and peptide cluster becomes less favorable in thick and stiff membranes, which favors the formation of larger aggregates, while the corresponding energy mismatch is reduced in soft and thin membranes. Our results present evidence that cross-*β* sheets of A*β*_25–35_ in anionic unsaturated lipid membranes can be re-dissolved by changing membrane properties to reduce domain mismatch.

## Introduction

A primary feature in the pathogenesis of Alzheimer’s disease is the deposition of insoluble fibrillar plaques in the extracellular space of brain tissue^1^. The major component of these plaques is a naturally existing peptide, the amyloid-*β* peptide (A*β*). A*β* undergoes conformational changes leading to aggregation and the development of neurodegenerative diseases, such as Alzheimer’s disease; however, the exact relationship between the two is still unclear^2^. While aggregation of proteins is an inherent part of aging to some extent^3^, increasing evidence has shown a link between the formation of plaques and the composition of surrounding brain tissue. Amyloid-*β* is a polypeptide consisting of 42 amino acids, which has a 10 amino acid long transmembrane segment, A*β*_25–35_, that is common to both the amyloid precursor protein (APP) and the full length A*β* peptide. While this short transmembrane segment is commonly used in the study of peptide interactions and partitioning in membranes, see for example^4–6^, it has also been reported to have neurotoxic properties^7–12^ and high tendency for aggregation and fibrillation^13–15^.

The secondary structure of a peptide and its interactions with the plasma membrane are essential in maintaining the function and integrity of the cell. By significantly altering its formation, the resulting changes can lead to the pathology of many diseases^16^. In Alzheimer’s disease, the monomeric A*β* peptides transition into long, peptide structures that form amyloid fibres. These fibres consists of arrays of *β* sheets running parallel to the long axis of the fibrils, the cross-*β* motif^17^, which are connected through steric zippers^1^. Formation of fibrils is believed to be initiated by a nucleation site, upon which further insoluble *β* sheet structures can grow upon. Membranes are believed to play a key role in this process as they may serve as such a nucleation point^1,18^.

While A*β* peptides are frequently reported in an extracellular location, A*β*_1–40_ and A*β*_1–42_ molecules were found to strongly interact with negatively charged lipids and to bind to anionic membranes^19–26^. A*β*_1–42_ and A*β*_25–35_ were found to be oriented parallel to the membrane surface or embedded in anionic lipid membranes^4–6,27–29^. At low concentrations of 3 mol%, the peptides that penetrated the bilayer were found in a *α*-helical monomeric conformation^6,29,30^. At higher concentration of 10 mol% and 20 mol%, cross-*β* sheet aggregates were reported to form^30^ (concentrations given are relative to the number of membrane lipids and indicate the peptide-to-lipid ratio). Through distortions created locally by embedded peptides, the membrane can influence how A*β*_25–35_ interacts to form aggregates^31–35^. As these forces are mediated by the membrane, they strongly depend on the membrane environment.

Although the amyloid cascade hypothesis is being questioned^36^, many anti-Alzheimer’s drugs attempt to prevent formation^37^, growth^38^ or reduce toxicity^39^ of amyloid fibres. Evidence has been presented that small molecules can also influence A*β* aggregation through mechanisms other than direct peptide binding. In particular, many of these molecules have known membrane interactions leading to changes in fluidity, thickness, and bending stiffness which could, in turn, influence peptide aggregation^31^.

As the membrane may play an integral role in plaque formation, we studied three membrane-active molecules: curcumin, acetylsalicyclic acid (ASA), and melatonin, with A*β*_25–35_. Curcumin has been speculated to be a protective agent to Alzheimer’s disease^40,41^, and has been shown previously to bind directly to the amyloid-*β* peptide^42^. ASA has been shown to affect membrane solubility of small molecules^43^, but may promote hemmorhaging rather than benefit for patients with Alzheimer’s Disease^44,45^. Finally, melatonin has been shown to interact directly with amyloid-*β* in membranes^46^, and slow cognitive impairment in patients with Alzheimer’s disease^47^.

**Figure 1:**
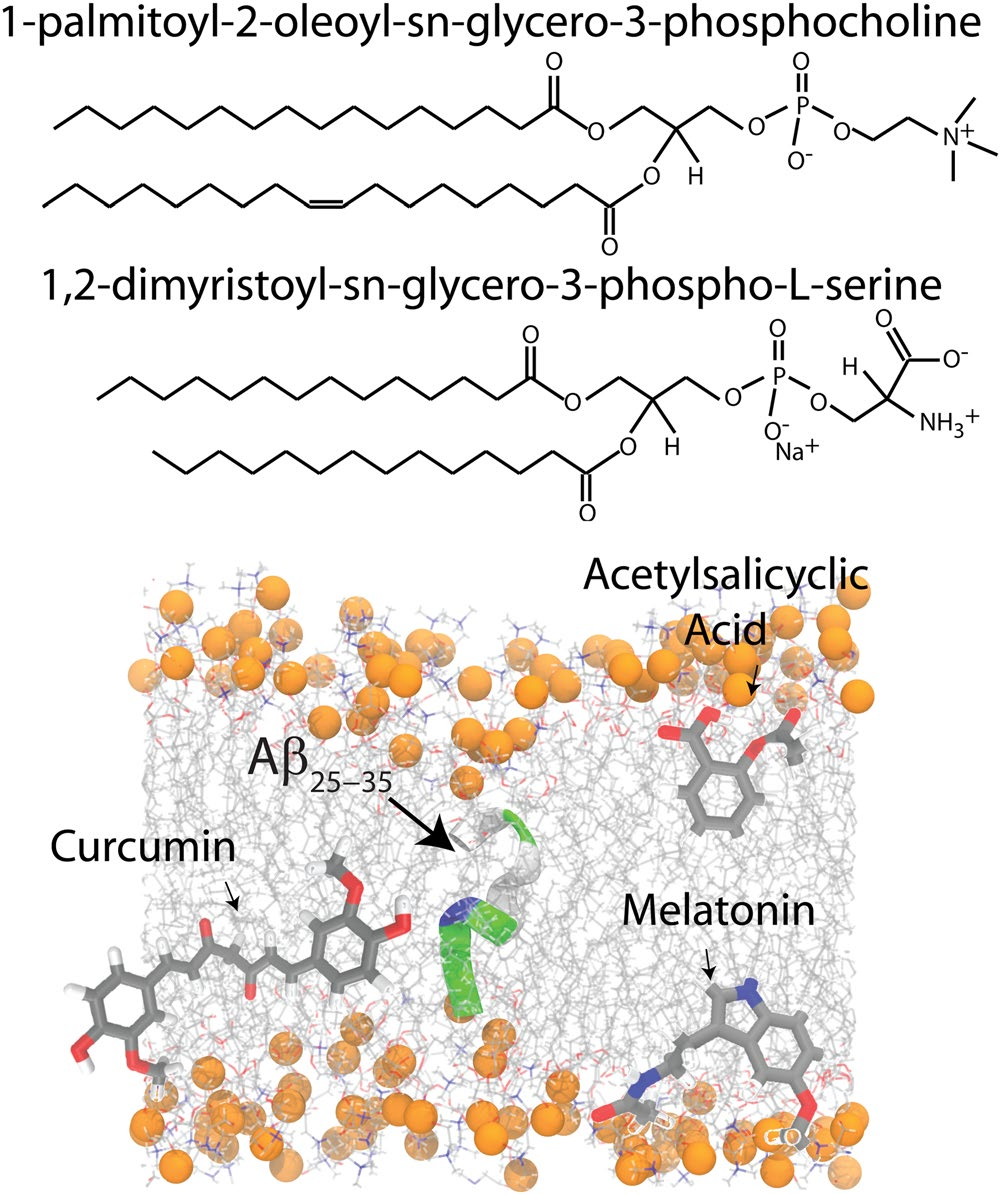
Schematic representations of 1-Palmitoyl-2-oleoyl-sn-glycero-3-phosphocholine (POPC), 1,2-Dimyristoyl-sn-glycero-3-phosphoserine (DMPS), Amyloid-*β*_25–35_ (C_45_H_81_N_13_O_14_S), acetylsalicylic acid (C_9_H_8_O_4_), melatonin (C_13_H_16_N_2_O_2_) and curcumin (C_21_H_20_O_6_).

Here, highly-oriented lamellar bilayers of POPC/DMPS (97/3 mol/mol%) were prepared on silicon wafers with or without the addition of 20 mol% A*β*_25–35_. The molecules are depicted in Fig. 1. At these high peptide concentrations, A*β*_25–35_ form aggregates consisting of cross-*β* sheets. We used scanning transmission x-ray microscopy, optical microscopy, atomic force microscopy, x-ray diffraction, UV-vis spectroscopy and MD simulations to quantitatively examine the effect of melatonin, ASA and curcumin on the amyloid aggregates. The molecules were added at concentrations of 5 mol% (drug-to-lipid ratio). We found that melatonin did not change membrane structure and did not have an effect on peptide clusters. ASA and curcumin, however, affected membrane properties, such as thickness and stiffness, which had significant effects on size and volume fraction of the A*β*_25–35_ clusters.

## Results

### Atomic Force Microscopy

**Figure 2:**
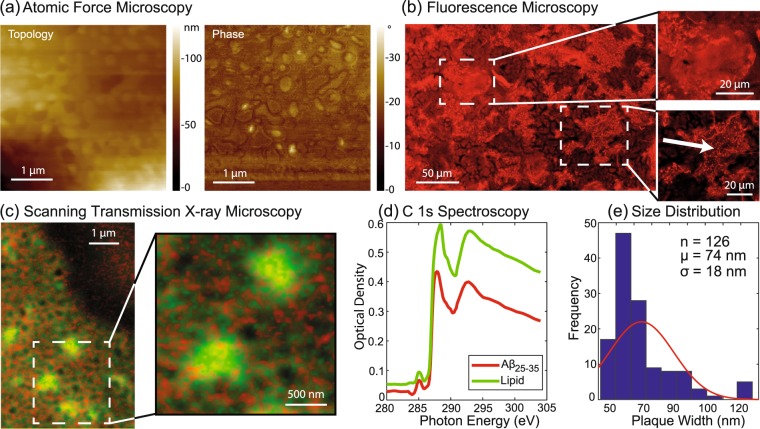
(**a**) AFM topology and phase images which show small, ~100 nm sized structures. These structures show strong autofluorescence in the optical microscopy image in part (**b**). The insets show evidence for larger peptide domains of some tens of *μ*m, which are formed by the aggregation of the small peptide clusters. (**c**) Scanning transmission x-ray microscopy images show clear evidence for peptide-rich clusters (peptides show in red). (**d**) Relative energy spectra of either lipid or peptide were used in order to discern the position and size of the peptide clusters on a membrane substrate. (**e**) Spatial analysis of the images reveals an average width of these plaques of 74 nm (with a standard deviation of 18 nm).

The topology of the multi-lamellar, solid supported membranes was investigated using an ezAFM+ from Nanomagnetics Instruments, as detailed in the Materials and Methods Section. Membranes were measured at a temperature of 24 °C. By operating the device in tapping mode, topology and phase pictures can be measured simultaneously. While the topology picture visualizes structures on the membrane surface, the phase picture highlights softer and stiffer regions of the bilayer. Figure 2(a) shows topology and phase image of of POPCP/DMPS + 20 mol% A*β*_25–35_. An area of ~4 × 4 *μ*m which was scanned with 2 *μ*m/s and a resolution of 512 × 512 pixel. The images show small, ~100 nm sized structures. The appearance of these structures in the phase image indicates changes in the membrane stiffness. In order to further clarify the origin of these structures, membranes were studied using fluorescence microscopy.

### Fluorescence Microscopy

Various amino acids are known to show autofluorescence. Peptides can emit a fluorescent signal when excited by an external light source. This property was used to identify peptide-rich areas in the bilayer. Fluorescence Microscopy was conducted using an Eclipse LV100 ND Microscope from Nikon, as detailed in the Materials and Methods Section. Samples were measured at a temperature of 24 °C. The corresponding image is shown in Fig. 2(b). Bright ~45 *μ*m sized regions are visible. Based on their strong autofluorescence, these regions were identified as peptide rich, in agreement with Tang *et al*.^30^ who have previously reported A*β*_25–35_ peptide plaques of tens of  *μ*m in POPC/DMPS bilayers using optical microscopy. The size of these regions also corresponds to the typical size of senile plaques observed in the brain tissue of Alzheimer’s patients. However, while some of these large plaques show a relatively uniform structure, the high resolution fluorescence microscope shows evidence that many of these plaques are composed of much smaller peptide rich clusters, about 100 nm in size, which correspond to the structures observed in AFM. The fluorescent images prove that these structures are indeed composed of peptides. The microscopy images also shed light on the formation of peptide aggregates: They suggest that small, ~100 nm peptide clusters form spontaneously in the unsaturated anionic membranes. These small clusters eventually fuse to form peptide plaques of several tens of *μ*m.

### Transmission X-ray Microscopy

Scanning transmission x-ray microscopy (STXM) was performed using the ambient STXM on beamline 10ID1 at the Canadian Light Source. Membranes were measured at a temperature of 24 °C. Results are shown in Fig. 2(c). Briefly, monochromated soft x-rays are focused to a 100 nm spot, the sample is placed at that focus, and images are formed by raster scanning the sample while synchronously recording the transmitted flux. Spectra are obtained by recording images at a set of photon energies, in this case, across the C 1s edge (280–305 eV). Transmitted signals are converted to optical density using the Beer-Lambert law ($OD=-\,\mathrm{ln}(I/{I}_{0})$), with *I*_0_ measured through the silicon nitride support where there is no sample. Lipids and peptides are distinguished spectroscopically by a 0.3 eV shift of the C 1s → π*C = *O* transition^48^, as shown in Fig. 2(d). Image sequences measured on the peptide-membrane aggregate are fit to reference spectra to form maps of the peptide and lipid components, which can be combined into color coded composites. Figure 2(c) reveals small peptide clusters associated with lipids. Spatial analysis (Fig. 2(e)) indicates the A*β*_25–35_ peptides form aggregates with an average size of 74 (18) nm in the anionic membranes, in excellent agreement with the results from AFM and optical microscopy. The molecular structure and composition of these peptide clusters was then studied using high resolution x-ray diffraction.

### X-ray Diffraction Signature of A*β* in Membranes

Highly-oriented lipid bilayers of POPC/DMPS (97:3 mol/mol) were prepared on silicon wafers with A*β*_25–35_ and the drugs curcumin, ASA, and melatonin. The membranes were hydrated and scanned in 97% RH conditions at a temperature of 30 °C.

**Figure 3:**
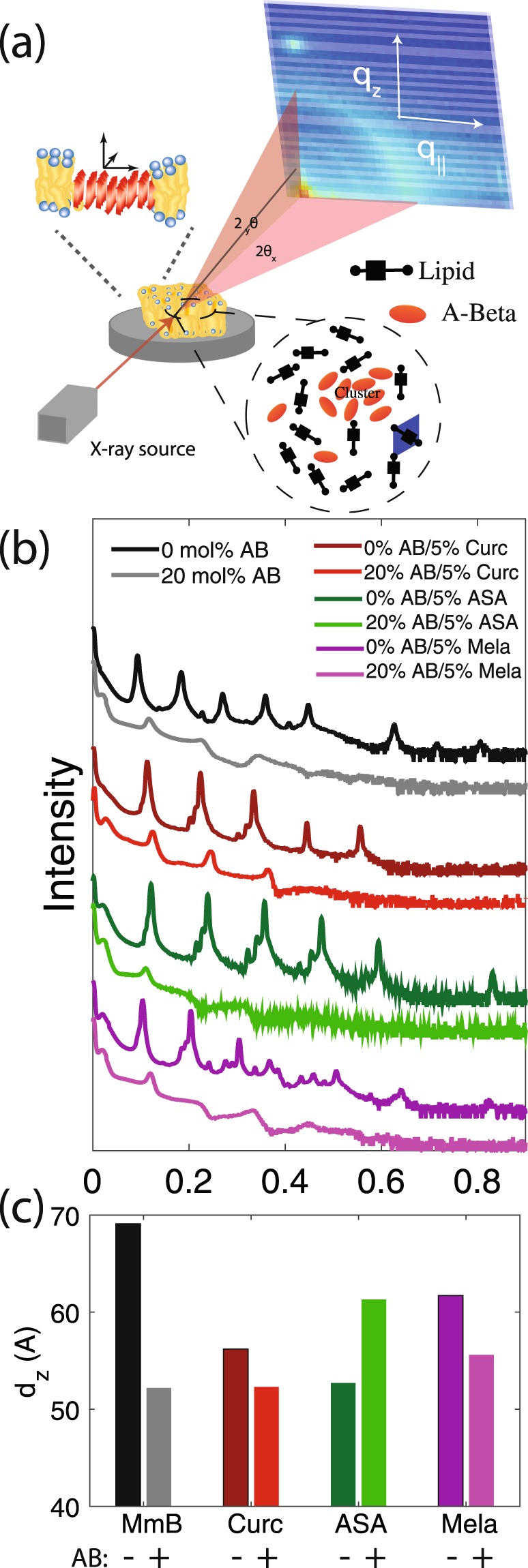
(**a**) Sketch of the experimental setup. (**b**) Out-of-plane (*q*_*z*_) x-ray diffraction for all membrane complexes in this study. (**c**) The lamellar spacing, *d*_*z*_ of membranes without and with peptide clusters. A decrease in *d*_*z*_ is observed both with the addition of small molecules and with the addition of peptide clusters. Errors on *d*_*z*_-values are 0.1 Å, as listed in Table 1.

Using high resolution x-ray diffraction, the in-plane (*q*_∥_) and out-of-plane (*q*_*z*_) structural features can be decoupled. The experimental setup is sketched in Fig. 3(a). Figure 3(b) presents out-of-plane diffraction data for pure POPC/DMPS bilayers without peptide and 20 mol% peptide added, and with the addition of 5 mol% curcumin, ASA and melatonin. A series of well developed Bragg peaks along *q*_*z*_ is the signature of well organized lamellar membranes. All membrane samples without A*β* show well developed Bragg peaks up to an order of 7. The addition of 20 mol% A*β*_25–35_ led to significant changes in the diffraction pattern: Bragg peaks were less pronounced and the number of higher orders was significantly reduced as a result of increased membrane bending, as will be discussed below. The lamellar spacing (membrane width + width of the hydration water layer), *d*_*z*_ is calculated from the spacings between the respective Bragg peaks using Bragg’s law, *d*_*z*_ = 2*π*/Δ*q*_*z*_. Results are plotted in Fig. 3(c) and listed in Table 1. The addition of A*β*_25–35_ significantly reduced *d*_*z*_ in pure membranes from 70 to 56 Å. A*β*_25–35_ was found to decrease the lamellar spacing also in the presence of 5 mol% curcumin and 5 mol% melatonin while 5 mol% ASA was found to lead to an increase in thickness.

**Table 1:** Structural parameters of membranes and peptide clusters for all membrane assays examined in this study.

Molecule (5 mol%)	A*β*_25–35_ (mol%)	*d*_*z*_ (Å)	*A*_*T*_ (Å^2^)	Lipid Tilt (°)	*H*	*d*_W_ (Å)	*d*_HH_ (Å)	Cluster Size, *L* (nm)	Cluster Volume Fraction
—	0	69.5 ± 0.1	22.6 ± 0.06	20.3 ± 0.9	96.8 ± 0.2	27.7 ± 0.8	41.8 ± 0.8	—	—
	20	55.2 ± 0.1	23.5 ± 0.1	18.3 ± 0.2	75 ± 1	16.2 ± 0.8	39 ± 0.8	21 ± 3	0.14 ± 0.02
Curcumin	0	56.2 ± 0.1	23.1 ± 0.04	20.3 ± 0.9	96.4 ± 0.1	20.4 ± 0.8	35.8 ± 0.8	—	—
	20	52.3 ± 0.1	23.8 ± 0.1	21.3 ± 1	88 ± 0.1	15.4 ± 0.8	36.9 ± 0.8	14 ± 10	0.04 ± 0.01
ASA	0	52.7 ± 0.1	22.9 ± 0.05	17.3 ± 1.5	97.5 ± 0.2	15.7 ± 0.8	37 ± 0.8	—	—
	20	61.3 ± 0.1	23.7 ± 0.1	25 ± 2	86.6 ± 1	20.3 ± 0.8	41 ± 0.8	106 ± 2	0.15 ± 0.02
Melatonin	0	61.7 ± 0.1	22.8 ± 0.05	17.3 ± 2	96.7 ± 0.1	22.3 ± 0.8	39.4 ± 0.8	—	—
	20	55.6 ± 0.1	23.6 ± 0.1	19.3 ± 0.2	87 ± 0.1	18 ± 0.8	37.6 ± 0.8	12 ± 2	0.16 ± 0.03

Parameters are given for pure POPS/DMPS membranes and POPC/DMPS + 5 mol% curcumin, ASA and melatonin. Given are the lamellar spacing, *d*_*z*_, the area per lipid tail, *A*_*T*_, the lipid tilt angle, the Herman orientation parameter, hydration water layer width, *d*_*W*_, membrane width, *d*_*HH*_, the average size of the peptide clusters, *L*, as determined from Scherrer’s equation, and the cluster volume fraction as determined from the ratio of the cross-*β*/lipid signals.

Membrane width, *d*_*HH*_, defined by the head-head distance, and the thickness of the hydration water layer, *d*_*w*_, were determined from Fourier transformation of the reflectivity data in Fig. 3, as detailed in a previous publications^6,29^. The corresponding values are given in Table 1. When peptides are embedded in pure POPC/DMPS and with the addition of curcumin and melatonin, there is a decrease in the thickness of the hydration water layer while the addition of ASA led to an increase in *d*_*w*_. By comparing the membrane widths, *d*_*HH*_ remained unchanged with the addition of melatonin (within the statistics of our experiment). The addition of ASA, however, led to an increase in membrane width while curcumin made the membranes thinner.

**Figure 4:**
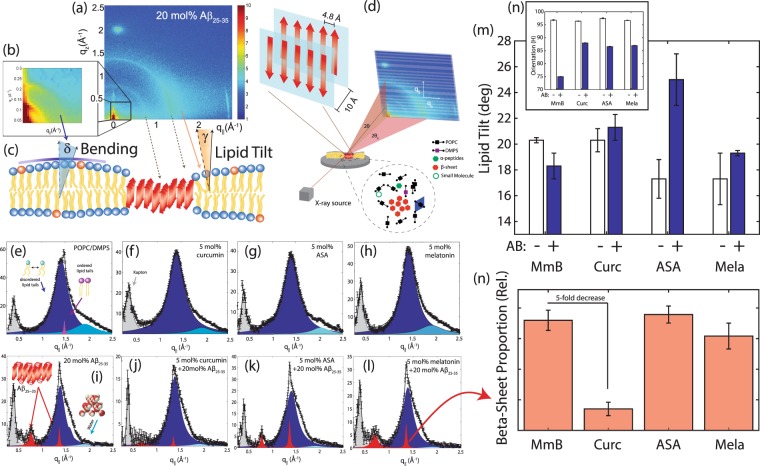
(**a**) Two-dimensional diffraction data for POPC/DMPS with 20 mol% A*β*_25–35_. (**b**) Shows the small angle region in magnification. Assignment of the different signals to the different components and membrane properties is shown in (**c**); the experimental setup is shown in (**d**). In-plane (*q*_||_) x-ray diffraction scans for all membrane complexes studied are shown in (**e**–**h**). For membranes without peptides, four signals are observed: (i) a peak at *q*_||_ ~ 0.4 Å^−1^ arising from the kapton windows of the x-ray chamber; (ii) a broad signal at *q*_||_ ~ 1.4 Å^−1^ from fluid lipid tails; (iii) A sharp signal at *q*_||_ ~ 1.5 Å^−1^ from gel phase lipids; and (iv) A broad feature at *q*_||_ ~ 1.85 Å^−1^ arising from amorphous water near lipid head groups. For membranes containing 20 mol% peptide, two additional peaks are observed at *q*_||_ ~ 0.7 Å^−1^ and *q*_||_ ~ 1.35 Å^−1^ which arise from A*β*_25–35_ peptides aggregating in cross-*β* sheets (**i**–**l**). The lipid tilt angle is shown in (**m**). (**n**) shows the relative amount of *β*-sheets. (Values are listed in Table 1).

In-plane diffraction results are shown in Fig. 4. Complete 2-dimensional data are shown for the POPC/DMPS assay containing 20 mol% A*β*_25–35_ in Fig. 4(a,b). The different signals can be assigned to different molecular components and membrane properties, as sketched in Fig. 4(c); Part d) shows a sketch of the experimental set up. Two-dimensional data were integrated and converted into line scans in Fig. 4(e–l). Membrane hydration water molecules organize at 3.4 Å with respect to each other, leading to a peak at *q*_∥_ = 1.85 Å^−1^. The broad peak of highest intensity centered around *q*_T_ ~ 1.4 Å^−1^ in fluid membranes is due to the packing of the lipid tails in the hydrophobic membrane core.

The area per acyl tail is obtained from the relation ${A}_{T}\,=\,8{\pi }^{2}/(\sqrt{(3)}{q}_{T}^{2})$, where *a*_*T*_ is the distance between acyl tails as calculated from ${a}_{T}=4\pi /(\sqrt{3}{q}_{T})$^30,49^; values for *A*_*T*_ are given in Table 1. The addition of A*β*_25–35_ led to a slight increase in lipid area in all assays. Additional small lipid signals are observed in the pure POPC/DMPS membranes in Fig. 4(e) at *q*_||_-values of 1.43 and 1.5 Å^−1^. These signals have been reported before^50–52^ and assigned to the organization of the lipid head groups within the lipid matrix. Peak positions are well described by a rectangular unit cell with dimensions *a* = 8.4 Å and *b* = 8.8 Å. We note that these signals disappear with the addition of curcumin, ASA and melatonin indicative of an increased disorder in membrane organization.

The average orientation of the lipid bilayers and the tilt of the lipid molecules can be determined by studying the angular dependence of the corresponding diffraction signals in the 2-dimensional x-ray intensity maps. The intensity at the lipid tail position was integrated as function of the azimuthal angle *γ*, to determine the average tilt angle of the lipid acyl chains. The corresponding values are listed in Table 1.

Figure 4(b) shows the small angle region around the reflectivity Bragg peaks in magnification. The corresponding intensity shows a circular pattern and was integrated over the meridian, *δ*, and analyzed using Hermans orientation function, as detailed in the Materials and Methods Section. Hermans function describes the degree or extent of orientation of the molecular axis relative to the membrane normal. Completely aligned would result in *f* = 1, randomly oriented in *f* = 0.25. Values for membrane curvature and lipid tail orientation are shown in Fig. 4(m,n) and also listed in Table 1. Bending of the bilayers and the average tilt angles of the lipid tails increase with peptide concentration, indicative of increasing bilayer distortions in the presence of peptides and peptide aggregates.

The signals at ~10 Å (*q*_||_ = 0.7 Å^−1^) and 4.7 Å (*q*_||_ = 1.35 Å^−1^) are the pattern of amyloid peptides forming cross-*β* amyloid sheets. The structure of a cross-*β* sheet is depicted in Fig. 4(d). The two reflections observed in the x-ray pattern correspond to inter-strand and inter-sheet distances of peptide chains^1,30^. The reflection at 1.35 Å^−1^ is indicative of extended protein chains running roughly perpendicular to the membrane plane and spaced 4.7 Å apart. The reflection at 0.7 Å^−1^ shows that the extended chains are organized into sheets spaced 10 Å apart. The signal at *q*_||_-values of ~0.4 Å^−1^ (marked in grey) stems from the Kapton windows of the humidity chamber and was, therefore, not included in the structural analysis.

The intensities of the peptide signals in Fig. 4(i–l) are proportional to the volume fraction of the different phases. The volume fraction of A*β*_25–35_ aggregates can be determined from the ratio of the integrated diffraction signals. Values are given in Table 1 and displayed in Fig. 4(n). While a volume fraction of aggregates of 14% was observed in pure POPC/DMPS, 15% were found with ASA and 12% with melatonin. A significantly lower percentage of 4% was found in the presence of curcumin. As 20 mol% of the bilayers are made of peptides, they should contribute 20% to the scattering signal. However, the scattering experiment is only sensitive to aggregated peptides. Therefore, a peptide signal of 15% means that 3/4 of the added peptides (out of the 20 mol% added), are organized in clusters while 1/4 still exist as monomers, either embedded or outside of the the bilayers. In the presence of curcumin, only 1/5 of all added peptides are found in clusters while 4/5 exist as monomers.

**Figure 5:**
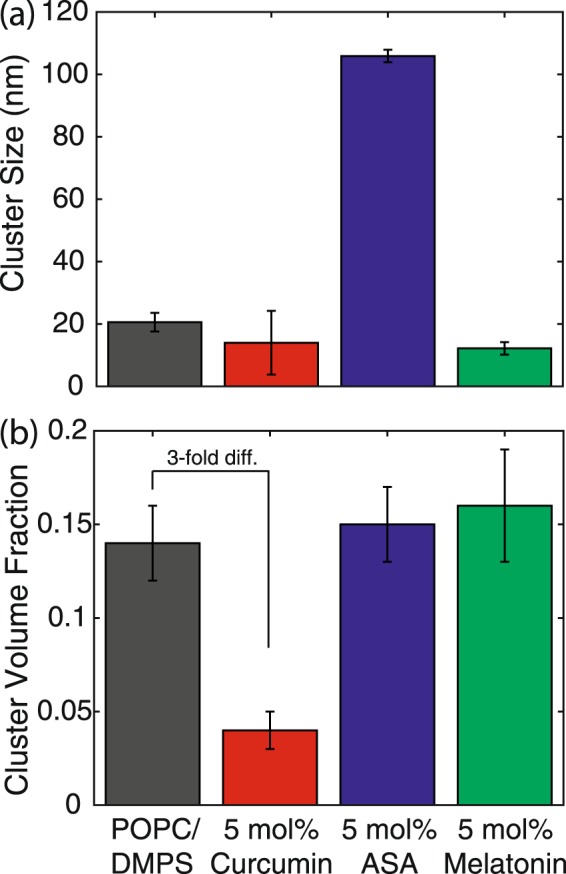
(**a**) The cluster size *L* is determined from the width of the peptide peak at *q*_||_ ~ 0.7 Å^−1^ using Scherrer’s equation. While melatonin and curcumin led to a slight decrease in cluster size, ASA significantly increased A*β* clusters. (**b**) The volume fraction of peptide clusters is calculated by integrating the area under the peptide peaks and normalizing by the integrated intensity of the lipid peak. Curcumin was found to cause as significant decrease in cluster volume fraction.

The sizes of the corresponding peptide aggregates can be estimated from the width of the corresponding diffraction peaks using Scherrer’s equation^53,54^. Cluster size and volume fraction are plotted in Fig. 5(a,b) (and also listed in Table 1. The size of peptide clusters was in the order of ~100 nm for POPC/DMPS in the microscope images in Fig. 2, while all cluster sizes determined by x-ray diffraction except for ASA were significantly smaller (pure membrane 21 nm, curcumin 14 mn, melatonin 12 nm). Although this could be the result of a general discrepancy in size determination using different techniques, it likely points to a domain sub-structure of the peptide clusters, *i*.*e*., that the 100 nm aggregates have grown from smaller nuclei, which then form domains of different orientation within the peptide clusters. X-ray diffraction would then give the width of the smaller domains, while the microscopical techniques are sensitive to the total size of the cross-*β* clusters.

### UV-vis Spectroscopy

The thioflavin T fluorescence (ThT) assay is commonly used for the detection of amyloid fibrils^55^. The ThT class of molecules has three distinct binding sites in A*β* peptides^56^. ThT binds to “cross-strand ladders” that are inherent in repeating side-chains interactions running across the *β*-strands within a *β*-sheet layer^57^ and is used to quantify the presence of cross-*β* sheets in liposome solutions with UV-visible spectroscopy to corroborate our previous experimental findings. In the presence of cross-*β* sheets, thioflavin T absorbs light at 456 nm.

**Figure 6:**
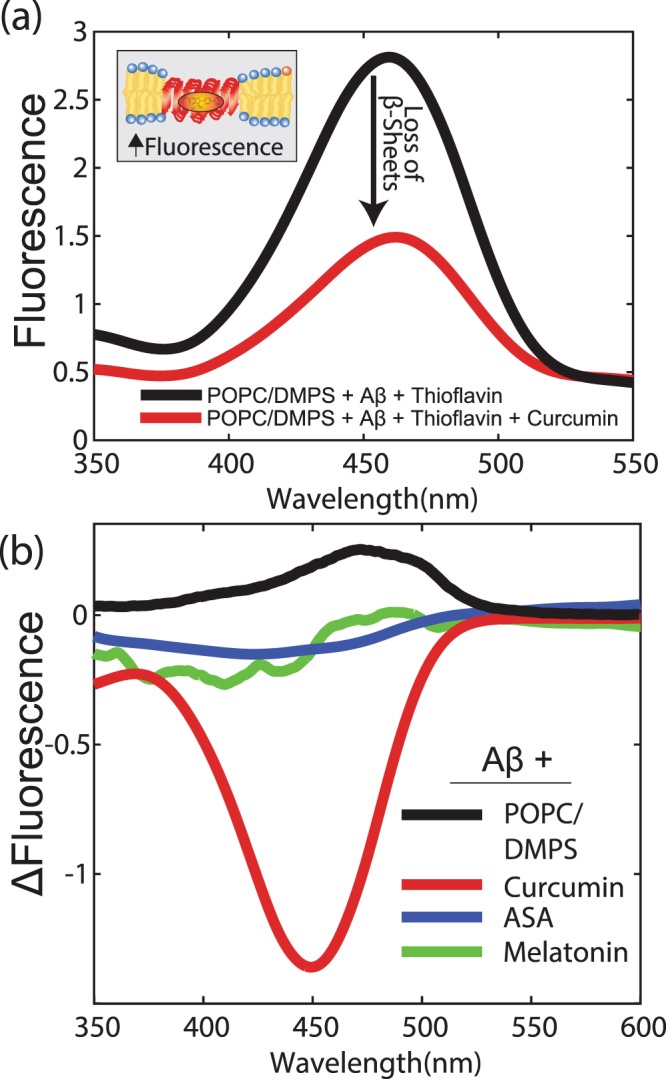
(**a**) Fluorescence of POPC/DMPS + Amyloid-*β* with Thioflavin T. The high fluorescence around 450 nm is the result of *β*-sheet formation. Fluorescence significantly decreases with the addition of curcumin. (**b**) Difference curves in UV signature for curcumin, ASA, and melatonin normalized to that of a pure POPC/DMPS bilayer with 20 mol% A*β*_25–35_. While there was no change in the amount of cross-*β* sheets for ASA and melatonin, the decrease in signal in the case of curcumin is indicative of *β*-sheet depletion. Pure POPC/DMPS is shown as a control for *β*-sheet formation.

We first prepared liposomes of POPC/DMPS (97:3 mol/mol%) and added 20 mol% A*β*_25–35_ and Thioflavin T to form peptide aggregates. The corresponding spectrum in Fig. 6(a) shows fluorescence at 456 nm, characteristic of cross-*β* sheets. After a stable fluorescence was reached, small volums of melatonin, ASA or curcumin were added to the solution. When adding curcumin, the fluorescence was found to decrease. Figure 6(b) shows the difference in fluorescence for the three molecules normalized to that of the pure POPC/DMPS matrix. No reduction in fluorescence was observed after the addition of melatonin or ASA. In assays with curcumin; however, the fluorescence decreased significantly, indicating a depletion of cross-*β* sheets.

### Molecular Dynamics Simulations

The effect of a single embedded A*β*_25–35_ molecule on bilayer structure, and the impact of curcumin, ASA and melatonin was investigated using all-atom MD simulations. Four 200 ns all-atom MD simulations were conducted with the CHARMM36 force field with the GROMACS 5.1.2. MD package. The peptide segment A*β*_25–35_ was obtained from RSCB (PDB: 1AKI) and simulated for 300 ns in bulk solution. 128-lipid (64 per leaflet) systems were prepared which mimicked experimental concentrations (124 POPC: 4 DMPS), and a modified InflateGRO package to insert the A*β*_25–35_ peptide into the center of the bilayer. Simulations were performed without and with each drug, solvated to *n*_*w*_ = 25 and charge neutralized. The system was then equilibrated by an NVT and NPT ensemble, and equilibrated as explained further in the Methods section. Simulations were run for 200 ns of which the last 10 ns was used for analysis.

**Figure 7:**
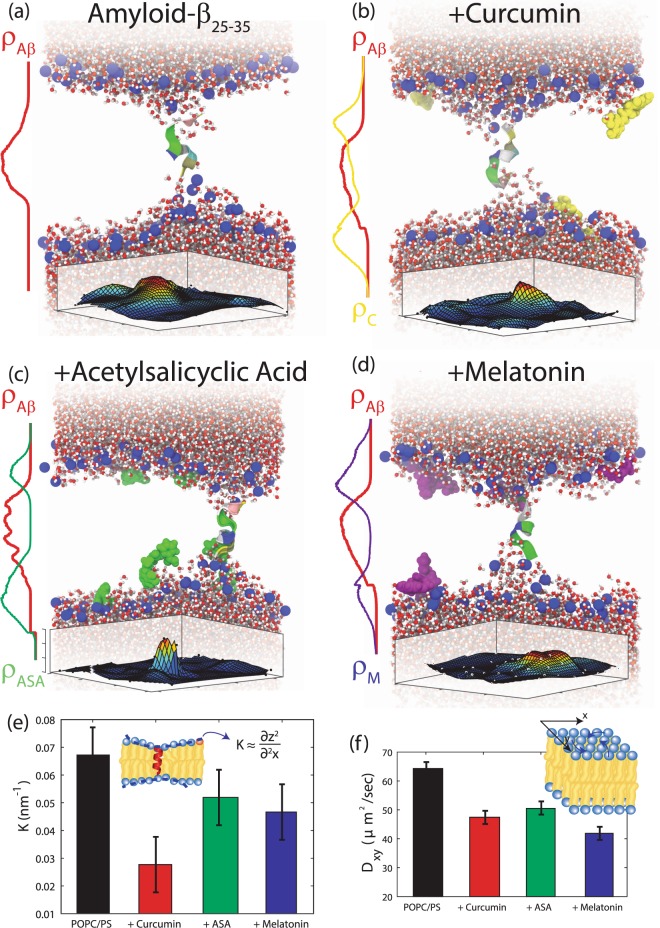
Snapshots of each lipid: A*β* system are shown, where the red line correspond to the relative electron density of the inserted peptide and the yellow, green, and purple lines correspond to the calculated electron density of curcumin (**b**), acetylsalicyclic acid (**c**), and melatonin (**d**), respectively. The 2-dimensional surface displays the positions of the phosphorus. (**e**) Membrane curvature as calculated from the MD assays. All drugs lead to a decrease in membrane curvature with curcumin to a pronounced decrease of ~60%. (**f**) Lateral diffusivity of the lipid molecules in pure POPC/DMPS + A*β*_25–35_ and in the presence of curcumin, ASA and melatonin. The presence of all drugs led to a decrease in lipid diffusivity.

Snapshots from the simulations are shown in Fig. 7 and show the positions of the different molecules in the bilayers. Electron density profiles were generated to highlight the position. The A*β*_25–35_ peptide in Fig. 7(a) was initially centered at *z* = 0 but then localized toward the acyl tails of either leaflet. This slightly tilted position is in agreement with the position determined experimentally from x-ray diffraction^6^. Peptide partitioning caused negative membrane curvature on both sides of the membrane such that the C- and N- terminals of the peptide were positioned in the head-tail interface. All three drugs were found to position in the head-tail interface of the membrane, in agreement with experimental models of bilayers with curcumin (Fig. 7(b))^41^, ASA (Fig. 7(c))^52^, and melatonin (Fig. 7(d))^46^. The positions of the phosphorous atoms in the lipid head groups is visualized as 2-dimensional surface and shows increased membrane bending around the position of the A*β*_25–35_ peptides.

The local membrane curvature, *K*, was calculated by fitting the positions of the lipid head groups with respect to the membrane normal and subsequent Monge parameterizations, as discussed in the Materials and Methods Section. Values for *K* are plotted in Fig. 7(e). All drugs led to a decrease in membrane curvature with curcumin causing a 60% decrease. A decrease of *K* and decrease in this local curvature is indicative of a softening of the membranes. From the lipid diffusion displayed in Fig. 7(f), all drugs led to a slight decrease in diffusivity.

## Discussion

As all drugs investigated in this study do not directly interact with the amyloid peptides but instead impact on membrane properties, it is likely that membrane-mediated interactions between the inserted proteins play a major role in the aggregation behaviour of A*β*_25–35_. A hydrophobic mismatch is created between peptide domains and lipids when the hydrophobic thickness of the transmembrane proteins does not match the equilibrium bilayer thickness, which causes each monolayer leaflet to distort in order to ensure that the entire hydrophobic region of the peptide is contained within the hydrophobic core of the membrane. As reported previously^30–33,58–61^, this mismatch can result in long-range attractive forces between the peptides.

Multi-lamellar, solid supported membranes were prepared for the AFM, fluorescence microscopy, transmission x-ray microscopy and x-ray diffraction experiments. In this type of sample preparation, lipids, peptides and small molecules are all added at the time of preparation. To rule out that the observed effects are the result of the sample preparation, small unilamellar vesicles in solution were prepared for the UV-vis measurements, and peptides and small molecules added later. These assays were used to study the effect of curcumin, ASA and melatonin on size and volume fraction of the cross-*β* peptide clusters. MD simulations were specifically used to investigate the effect of insertion of a single peptide on the curvature of the bilayers to better understand the early stages of aggregate formation and derive a theoretical model.

A simple free energy model based on the concept of hydrophobic mismatch could be used to understand the observed effects of curcumin, ASA and melatonin on the size of the A*β*_25–35_ clusters. Regarding the clusters as a phase separated domain, their free energy can be written as the sum of contributions from the bulk free energy gain and interface energy (line tension). Assuming the aggregates are circular, the free energy of the clusters bilayer can be written in terms of the cluster radius *R* as,

1 $${\rm{\Delta }}G=-\,\pi {R}^{2}{g}_{0}+2\pi R\sigma ,$$

where *g*_0_ and *σ* are the free energy density (free energy per unit area) and line tension of the A*β*_25–35_ domain, respectively. For a binary system composed of phase separated domains, the size of the minority domains is dictated by the interfacial tension or line tension of the domains. In particular, a smaller interfacial or line tension would lead to a smaller domain size. The line tension between a bilayer membrane and a hydrophobic insertion depends on the hydrophobic mismatch. Specifically, the line tension is mainly governed by the thickness deformation energy of the bilayer near the hydrophobic insertion, $\sigma =\frac{1}{2}K{u}^{2}$, where *u* = *w* − *w*_0_ is the difference between the bilayer thickness w and the hydrophobic length *w*_0_, and $K=\sqrt{2}\sqrt[4]{{K}_{t}^{3}{K}_{b}/{w}^{6}}$ is the “spring constant” induced by the membrane deformation^62,63^. The hydrophobic thickness is determined by the thickness of the A*β*_25–35_ clusters, which is a constant for the different samples. Therefore the line tension of the A*β*_25–35_ clusters depends on the thickness of the bilayer as $\sigma \propto \frac{{(w-{w}_{0})}^{2}}{w(\mathrm{3/2)}}\sim {(w-{w}_{0})}^{2}$ if we assume that the thickness change of the membranes is small. From this thickness dependence and using the values of the bilayer thickness obtained from the experiments, we can conclude that the line tension of the clusters following the order of *σ*_*Curcumin*_ < *σ*_*Melatonin*_ < *σ*_*ASA*_. This ordering of the line tension is consistent with the observed size distribution of the A*β*_25–35_ aggregates.

In the case of melatonin, the membrane properties did not change and as a consequence, melatonin did not have a measurable effect on the amyloid peptide clusters. ASA led to a thickening and stiffening of the membrane thereby increasing the hydrophobic mismatch and making this interface energetically less favorable. As a result, the addition of ASA led to the formation of larger domains; however, the total amount of cross-*β* sheets was unchanged. This argument can be illustrated as follows: two domains of radius *r* have a total area of 2 × *πr*^2^ and a circumference of 2 × 2*πr*. A larger domain with the same area would have a radius of $r=\sqrt{2}r$ and a circumference of $2\pi (\sqrt{2}r)$, smaller than two smaller domains. At the same time lipid tilt has to increase in order to adapt to the larger hydrophobic mismatch, as observed in the structural data.

**Figure 8:**
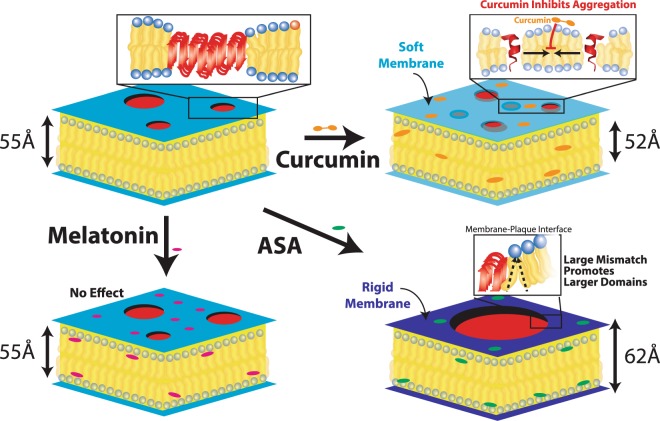
Summary of the experimental and computational findings. A*β*_25–35_ forms clusters in anionic unsaturated membranes made of POPC/3 mol% DMPS, which show the characteristic cross-*β* pattern. There is a hydrophobic mismatch between lipid and peptide domains. Melatonin does not affect membrane properties and does not have an observable effect on peptide aggregates. ASA leads to a thickening and stiffening of the membranes in the presence of A*β* peptides, which increases the mismatch energy and favours the formation of larger domains. Curcumin on the other hand, leads to a thinning and softening of the membranes, which results in a significant reduction of the amount of cross-*β* sheets.

Curcumin, on the other hand, was found to lead to a thinning and softening of the membranes, which significantly reduces the energy cost in Eq. (1) and favors dissolution of the peptide domains. Lipid tilt was unchanged and the membrane order parameter, *H*, points to small membrane bending, consistent with the idea of relatively flat bilayers. Thus the results point at a membrane mediated mechanism for the formation of A*β*_25–35_ peptide clusters in unsaturated anionic lipid membranes. The findings are summarized Fig. 8.

## Conclusion

Model unsaturated anionic neuronal membranes were studied at high concentrations of A*β*_25–35_, the transmembrane segment of the Amyloid-*β* peptide. Multi-lamellar, solid supported membranes were prepared for these experiments. The peptides form peptide clusters at high peptide concentrations of 20%, which show the cross-*β* pattern also observed in the plaques of Alzheimer’s patients. Formation of peptide clusters was confirmed by atomic force microscopy, fluorescence microscopy and transmission x-ray microscopy. These techniques present evidence for small, ~100 nm sized clusters, which organize and fuse to form larger plaques of tens of *μ*m. In order to test the effect of membrane properties on peptide cluster formation, melatonin, ASA and curcumin were added to the membranes. These molecules are membrane active and have been speculated to affect amyloid aggregation.

From x-ray diffraction, we find that melatonin does not change membrane properties and did not have an observable effect on the peptide clusters. ASA led to a thickening and stiffening of the membranes, which resulted in the formation of larger peptide clusters. The addition of curcumin led to a thinning and softening of the membranes, resulting in a significant decrease of the amount of the cross-*β* sheet signal indicative of a dissolution of the A*β* clusters by 70%. Results were confirmed using UV-vis spectroscopy and the Thioflavin T assay, where curcumin led to a significant reduction in the *β* sheet signal.

In all-atom MD simulations, the addition of A*β*_25–35_ peptides to the membranes led to an increase of local curvature related to membrane bending around peptide inclusions. Curcumin was found to reduce local curvature. We therefore hypothesize that the hydrophobic mismatch between lipid and peptide domains and the stiffness of the membranes play an important role in A*β*_25–35_ peptide aggregation in these membranes. Molecules which increase the hydrophobic mismatch, such as ASA, may increase the size of peptide aggregates, while molecules which decrease the membrane width can dissolve clusters.

## Materials and Methods

### Preparation of Highly-Oriented Multi-Lamellar Membranes

Highly oriented multi-lamellar membranes were prepared on single-side polished silicon wafers. 100 mm diameter, 300 *μ*m thick silicon (100) wafers were pre-cut into 1 × 1 cm^2^ chips. The wafers were first pre-treated by sonication in dichloromethane at 310 K for 30 minutes to remove all organic contamination and leave the substrates in a hydrophobic state. Each wafer was thoroughly rinsed three times by alternating with ~50 mL of ultrapure water and methanol.

Solutions of 1-palmitoyl-2-oleoyl-*sn*-glycero-3-phosphocholine (POPC) and 1,2-dimyristoyl-*sn*-glycero-3-phospho-L-serine (DMPS) at a concentration of 20 mg of lipid per mL of solvent were dissolved in a 1:1 chloroform: 2,2,2-trifluoroethanol (TFE) solution. The A*β* peptides were prepared by pre-treatment with trifluoroacetic acid (TFA) to disaggregate the peptide, as described by^64^. This pretreatment included dissolving the peptide in a 1 mg/mL solution of TFA, sonicating with a tip sonicator for four three second intervals, and then removing the solvent through evaporation using nitrogen gas then placed in a vacuum for 30 minutes at 298 K to remove any traces of TFA. The peptide was then redissolved in a 20 mg/ml solution of 1:1 TFE:chloroform. Stock solutions of melatonin, ASA and curcumin were prepared at a concentration of 20 mg/mL as well.

Each solution was vortexed until the solution was homogeneous. The POPC, DMPS, small molecules, and peptide solutions were then mixed in appropriate ratios to produce the desired membrane samples for the experiment. Schematics of the POPC, DMPS and A*β*_25–35_ molecules are shown in Fig. 1. The mol%-values given refer to the number of peptide and drug molecules per lipid molecule and indicate the peptide-to-lipid and drug-to-lipid ratios.

The temperature of the main transition in pure POPC is −2 °C. Care was taken to prepare the membranes at elevated temperatures, in the fluid phase of the lipid bilayers. The tilting incubator (VWR Incubating Rocker/3-D Rotator Waver) was heated to 313 K and the lipid solutions were placed inside to equilibrate. 65 *μ*L of lipid solution was applied on each wafer, and the solvent was then allowed to slowly evaporate for 10 minutes (speed 15, tilt of 1), such that the lipid solution spread evenly on the wafers. After drying, the samples were placed in vacuum at 313 K for 12 hours to remove all traces of the solvent. The bilayers were annealed and rehydrated before use in a saturated K_2_SO_4_ solution which provides ~97% relative humidity (RH). The hydration container was allowed to equilibrate at 293 K in an incubator. The temperature of the incubator was then increased gradually from 293 K to 303 K over a period of ~5 hours to slowly anneal the multi-lamellar structure. This procedure results in highly oriented multi-lamellar membrane stacks and a uniform coverage of the silicon substrates.

About 3,000 highly oriented stacked membranes with a thickness of ~10 *μ*m are produced using this protocol. The high sample quality and high degree of order is a prerequisite to determine in-plane and out-of-plane structure of the membranes separately, but simultaneously. Table 1 lists all samples prepared for this study.

### Scanning Transmission X-ray Microscopy

STXM measurements were performed using the ambient STXM on beamline 10ID1 at the Canadian Light Source (CLS, Saskatoon, SK, Canada). As measured transmission images were converted to optical density (OD) images using the Beer-Lambert Law, $OD=\,\mathrm{ln}({I}_{/}I)$, where *I*_0_ is the incident photon flux measured through a blank area of the silicon nitride window and *I* is the photon flux through an area where the sample is present. Since there was only partial coverage of the sample by the lipid peptide aggregates, lipid bilayer areas were identified using the difference in *OD* images measured at 288.8 eV (peak C 1s signal from lipids) and at 280 eV (below the C 1s onset). A full C 1s image sequence from 280 eV to 340 eV was then measured on areas typically 10 *μ*m × 10 *μ*m using 100 nm steps and with the STXM beam defocused to 100 nm to match the step size. Radiation damage to lipids is a concern^65^ and the reason why we chose to use defocused beam sizes, which reduced the dose by 10-fold relative to the use of a fully focused ~30 nm spot. The membrane sample was measured by x-ray diffraction before and after the STXM measurements and no structural differences were observed within the experimental resolution. The photon energy step size was 0.10 eV from 284 eV to 290 eV and 0.25 eV or larger outside this region. Axis2000 (available at http://unicorn.mcmaster.ca/aXis2000.html) was was used for stack alignment, conversion to *OD*, and singular value decomposition mapping using lipid and peptide spectra taken from the data measured in this study.

### Atomic Force Microscopy

Atomic Force Microscopy was conducted in the Origins of Life Laboratory at McMaster University using an Nanomagnetics ezAFM+. The instrument uses a FPGA based digital feedback control and is able to scan an area of 40 × 40 *μ*m with a maximal hight difference of 4 *μ*m. Samples are taped on a steel plate, which is then magnetically mounted on a 38 mm motorized XY-stage. A digital microscope with a field of view of 390 × 230 *μ*m can be focused either on the cantilever or sample surface and allows aligning the specimen with respect to the cantilever tip.

All presented images were recorded in non-contact (tapping) mode. The instrument was equipped with a Point Probe Plus Non-Contact Long-Cantilever Reflex Coating (PPP-NCLR) probe with an guaranteed tip radius of less than 10 nm and a resonance frequency of 190 kHz. Operating the instrument in tapping mode offers a simultaneous measurement of the topology and surface phase. First an area on the sample surface was chosen by the digital microscope and the motorized stage. Afterwards a coarse scan was performed over an area of 20 × 20 *μ*m with a scanning speed of 10 *μ*m/s. Flat areas were identified on this scan and rescanned with a scanning speed of 2 *μ*m/s at a resolution of 512 × 512 px. Topology data was processed using an auto-plane correction to correct the sample tilt, a scar and a horizontal line correction to correct artifacts. All data was processed using the NanoMagnetics Image Analyzer Software (version 1.4). Phase pictures were corrected by using the spot removal tool.

### Epi-Fluorescent Microscopy

Fluorescent Microscopy was conducted using an Eclipse LV100 ND Microscope from Nikon in the Origins of Life Laboratory at McMaster University. The instrument is equipped with a Tu Plan Fluor BD 50× objective with an numerical aperture of 0.8. Images were recorded using a Nikon DS-Ri2 Camera with a resolution of 4908 × 3264 pixel and a pixel-size of 7.3 × 7.3 *μ*m. The camera is mounted via a 2.5× telescope to the microscope. All presented images were recorded in episcopic illumination mode using a halogen lamp. Due to the high numerical aperture, the objective has a small depth of focus between 0.7 *μ*m and 0.9 *μ*m. In order to record a uniform sharp image, the Nikon control software (NIS Elements, Version 4.60.0) was used to record an Extended Depth of Focus (EDF) image by combining multiple images with different focal planes.

The presented data are a combination of two EDF pictures. First, a bright-field image has been recorded. Second, a fluorescent picture was taken. A B-2A longpass emission filter cube was used with an excitation wavelength of 450–490 nm and a long-pass analyzing filter with an barrier wavelength of 520 nm. Due to autofluorescence, peptides, such as the analyzed amyloid-*β* peptide, light up in the fluorescent picture. Phospholipids, on the other hand, barely emit a fluorescent signal. Hence, one can easily identify amyloid-*β* enriched regions on the fluorescent image. To locate the amyloid-*β* clusters on the sample surface, both a bright-field image and a fluorescent image were combined.

### UV-Visible Spectroscopy

UV-Visible spectroscopy was conducted using a Nanophotometer (IMGEN). 400 *μ*L of small unilamellar vesicles (SUVs) were prepared in water by probe sonication, preserving the POPC/DMPS (97:3 mol/mol%) ratio at a concentration of 5 mg/mL of lipid. The samples were kept in ice-bath during sonication to prevent solvent evaporation, and the probe was pulsed over an hour at 20,000 Hz to prevent the sloughing off of the titanium tip^66^. 1.8 mg of A*β*_25–35_ was then added to mimic a concentration of 20 mol% to replicate experimental aggregation conditions. The solutions was then transferred to a cuvette and used as blanks. Complete wave scans were taken from 200 nm to 800 nm, and fluorescence of thioflavin T at a wavelength of 456 nm was monitored^55,67^.

Because ThT can accelerate deposition of A*β* peptides^68^ and other amyloids^69,70^, experiments without the presence of drugs were conducted over a period of 24 hours to check for aggregation. Samples of A*β*_25–35_ were mixed in a 1.5 mL flask and kept in a shaking incubator at 37 °C. Aliquots were taken and placed in a cuvette at each time point. ThT was then added to the aliquot and a measurement was taken. ThT was found to induce aggregation, which plateaued out after 12 hours. All measurements in the manuscript were therefore conducted 12 hours after ThT deposition. When the plateau was reached, a small concentrated 100 *μ*L volume of a dissolved drug in water was added to the cuvette to minimize the decrease in measured emission from the increase in volume. The concentrations of these dissolved drugs were chosen to ensure the total solution had 5 mol% of drug to be in agreement with experiment. Full wave scans were then taken every 30 seconds for the next 10 minutes. As changes in Thioflavin T signal happened apparently instantaneously, we were not able to detect a change in signal temporally within the capabilities of our spectrophotometer.

### X-ray Diffraction

X-ray diffraction data was obtained using the Biological Large Angle Diffraction Experiment (BLADE) in the Laboratory for Membrane and Protein Dynamics at McMaster University. BLADE uses a 9 kW (45 kV, 200 mA) CuK*α* Rigaku Smartlab rotating anode at a wavelength of 1.5418 Å. Both source and detector are mounted on movable arms such that the membranes stay horizontal during the measurements. Focussing multi-layer optics provides a high intensity parallel beam with monochromatic X-ray intensities up to 10^10^ counts/(s × mm^2^). This beam geometry provides optimal illumination of the solid supported membrane samples to maximize the scattering signal. By using highly oriented membrane stacks, the in-plane (*q*_||_) and out-of-plane (*q*_*z*_) structure of the membranes can be determined separately but simultaneously.

The result of such an x-ray experiment is a 2-dimensional intensity map of a large area (0.03 Å^−1^ < *q*_*z*_ < 1.1 Å^−1^ and 0 Å^−1^ < *q*_||_ < 3.1 Å^−1^) of the reciprocal space. The corresponding real-space length scales are determined by *d* = 2*π*/|*Q*| and cover length scales from about 2.5 to 60 Å, incorporating typical molecular dimensions and distances. These 2-dimensional data are essential to detect and identify signals from bilayers and peptides and determine orientation of the molecules. All scans were carried out at 28 °C and 97% RH. The membrane samples were mounted in a humidity controlled chamber during the measurements. The membranes were hydrated by water vapour and allowed to equilibrate for 10 hours before the measurements to ensure full re-hydration of the membrane stacks.

The degree of orientation of the bilayers and lipid tails within the membrane samples was determined from the 2-dimensional x-ray maps. The intensity as a function of *Q* and angle *γ* from the *q*_||_ axis was used to determine orientation of lipid tail signals. Pixels within a wedge of the reciprocal space map, defined by *γ* and *γ*_*step*_ (where *γ*_*step*_ = 2°), were integrated as a function of $Q={({q}_{z}^{2}+{q}_{\parallel }^{2})}^{\mathrm{1/2}}$ and normalized by pixel count at each *Q*. *γ* varied from 30° to 90° for the sample with 20 mol% peptide to capture peptide signals. Data from *γ* < 30° was not included due to high absorption at low angles^71^. The integrated *I*(*Q*, *γ*) could be fit with Lorentzian functions. By calculating the area under the Lorentzian fits, *I*(*γ*) was determined and fit with a Gaussian distribution.

To determine the degree of orientation of membranes in the stack, the intensity as a function of the meridional angle *δ* was determined. The intensity was integrated around the second Bragg peak, at *Q* ≈ 0.22 Å^−1^, from 18° < *δ* < 40°. *δ* < 18° was not used in order to avoid contributions from diffuse scattering^72^. The second Bragg peak was chosen as diffuse scattering was weaker than the first Bragg peak. Pixel density at low-*Q* was too low to calculate *I*(*Q*, *δ*) as with the peptide samples, so *I*(*δ*) was calculated by direct summation of pixels within *δ* and *δ*_*step*_ (where *δ*_*step*_ = 2°), and within *Q* and *Q*_*step*_, where the *Q*-range was chosen to include only scattering from the second Bragg peak. *I*(*δ*) was fit with a Gaussian distribution centred at *δ* = 0, which was then used to calculate the degree of orientation using Hermans orientation function:

2 $$f=\frac{3\langle {\cos }^{2}\,\delta \rangle -1}{2}.$$

### Molecular Dynamics Computer Simulations

All simulations were run in-house on MacSim, A GPU-accelerated workstation containing 20 physical Intel XeonCPU cores and two GeForce GTX 1080 graphics cards, totalling to 5120 CUDA cores. The A*β*_25–35_ peptide was taken from PDB 1QWP and equilibrated using typical parameters, in the presence of 1000 water molecules to reduce structural rigidity for 300 ns. The peptide was then desolvated and the structure was re-inserted into bilayer patches.

A system containing 124 POPC and 4 DMPS lipids evenly sectioned across each leaflet. The lipid topologies were taken from the CHARMM-GUI builder. The system was equilibrated at high hydrations (25 waters per lipid) for 200 ns before A*β*_25–35_ was added to the center of the bilayer by a modified InflateGRO algorithm for multicomponent bilayers. Topologies for all systems were generated with the CHARMM General Force Field (CGenFF) program. All simulations were performed using the GROMACS 5.1.2 software package^73,74^, utilizing the CHARMM36 force field. All simulations used a 2 fs time step, a periodic boundary condition applied in all directions, a short-range van der Waals cutoff of 1.2 nm, the particle-mesh Ewald solution for long-range electrostatics^75^, and the LINCS algorithm for determination of bond constraints^76^. A Nose-Hoover thermostat at 28 °C (with a time constant of *τ*_*t*_ = 0.5 ps) was used for temperature coupling^77^, while a Parrinello-Rahman semi-isotropic weak pressure coupling scheme was used to maintain a pressure of 1.0 bar (with a time constant of *τ*_*p*_ = 1 ps)^78^. The position of the A*β*_25–35_ was restrained during volume (NVT) and pressure (NPT) equilibration to avoid free space bias as systems were reduced. Restraints were removed during 200 ns simulation.

Calculating the membrane curvature from MD simulations is not as well-defined, as defining the surface which is being “curved” from a reference plane is difficult to define. For this reason, we used Monge parameterization, in which the reference plane is defined as the average distance along the membrane normal where the overall density is at minimum, i.e. the bilayer center (*d*_0_). The surface is defined by the distance *d*, from the surface to the center. If *δ* = Δ*d* = *d* − *d*_0_ at position *r*(*x*, *y*), we obtain the form *δ*(*r*). Thus, the curvature *K* within Monge gauge can be calculated by the relation,

3 $$K=\nabla \cdot {(\frac{\nabla \delta (r)}{\sqrt{1+{(\nabla \delta (r))}^{2}}},)}^{\nabla h\ll 1}\delta (r)$$

where ∇ and Δ are the nabla- and Laplace-operator on the reference plane, respectively. The use of ∇*δ* uses the small gradient approximation. From this, we are able to define the local curvature at the point of maximum deformation as a function, *f*(*x*), due to the presence of A*β*_25–35_ on either leaflet, *P*. If we imagine an external circle force applied on the bilayer, then a line bisecting the center of the force and point *P* can be defined as *p*_1_*(x)*, and a second line bisecting the center of the force at some point away from *P* can be defined as *p*_2_*(x)*. The intersection of these lines must satisfy *p*_1_(*x*_*c*_, *y*_*c*_) = *p*_2_(*x*_*c*_, *y*_*c*_) to set up the equation for the touching curvature of the force, *ρ*. Assuming that the gradient term of the surface *f* ′ is low,

4 $${\rho }^{2}={({x}_{c}-{x}_{0})}^{2}+{({y}_{c}-{f}_{0})}^{2}=\frac{{\mathrm{(1}+{{f^{\prime} }_{0}}^{2})}^{3}}{f{\text{'}^{\prime} }_{0}^{2}}$$

and

5 $$K=\frac{1}{\rho }=\frac{f^{\prime} (x)}{\sqrt{1+f^{\prime} {(x)}^{2}}}\approx f^{\prime\prime} (x\mathrm{).}$$

By imposing the positions of the lipid head groups of either leaflet onto a 2-dimensional plane along the membrane normal to a quadratic equation of the form, *f*(*x*) = *ax*^2^ + *bx* + *c*. The differentials give rise to calculating the surface curvature, *K*^79^.

## Data Availability

All data are included in the manuscript.
